# Failing on Both Sides of the Infarct: Lipid-Lowering Therapy and the Delivery Gap

**DOI:** 10.1016/j.jacadv.2026.103189

**Published:** 2026-09-23

**Authors:** Richard V. Milani, Carl J. Lavie

**Affiliations:** a Center for Healthcare Innovation, Sutter Health, San Francisco, California, USA; b John Ochsner Heart & Vascular Institute, Ochsner Clinical School, The University of Queensland, New Orleans, Louisiana, USA

**Keywords:** acute myocardial infarction, clinical decision support, cognitive load, lipid-lowering therapy, LDL cholesterol, prevention, therapeutic inertia

**Figure:**
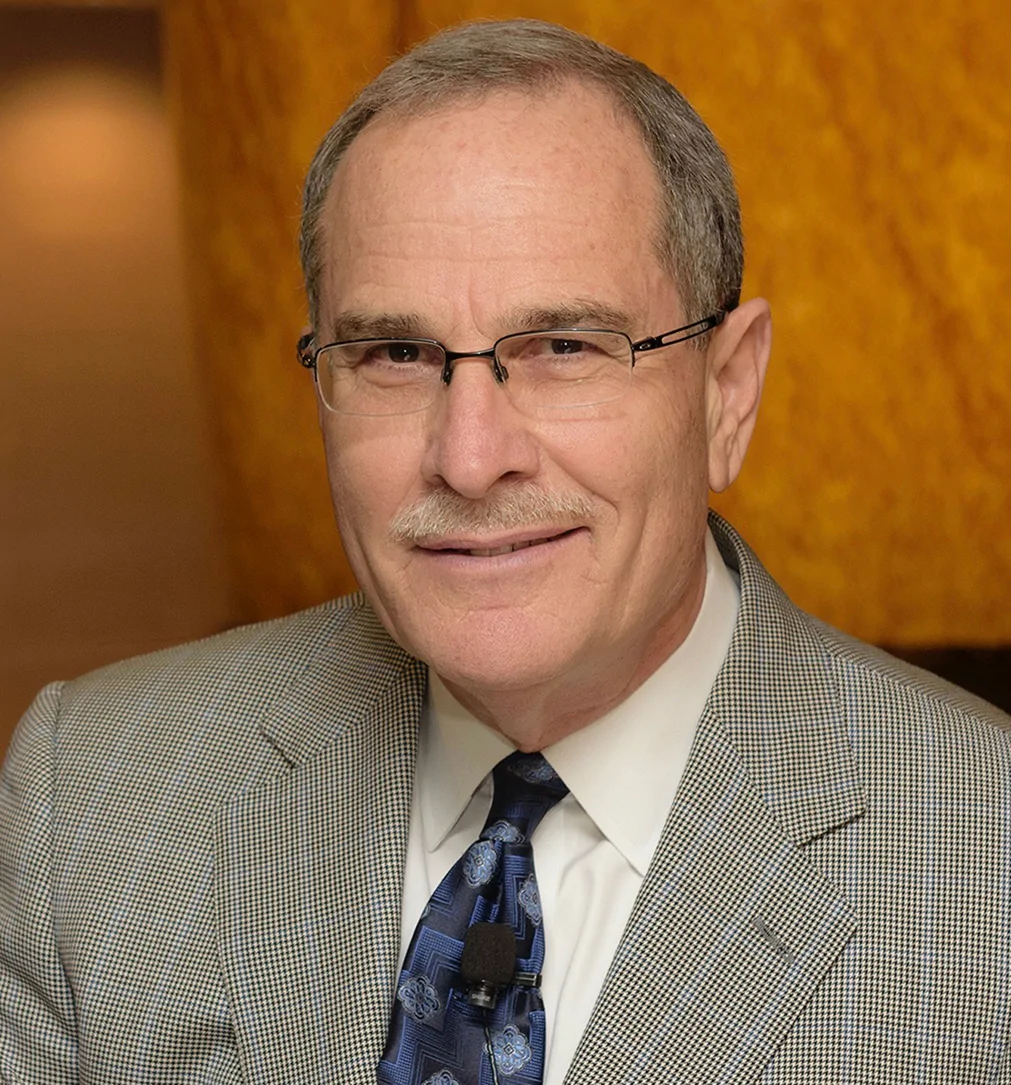

Few propositions in cardiovascular (CV) medicine are as thoroughly settled as the case for lowering low-density lipoprotein cholesterol (LDL-C). It is causal, it is dose-dependent, and it is treated with inexpensive, well-tolerated agents. And yet, for most patients who suffer an acute myocardial infarction (AMI), the infarction itself is the moment the health system first acts on a risk that was visible, measurable, and modifiable for years beforehand. In this issue of *JACC: Advances*, Essa et al^1^ hold a mirror to that failure, and the reflection is unflattering on both sides of the event.

Drawing on 1,848 patients hospitalized with type 1 AMI in the Yale NCDR CathPCI Registry between 2018 and 2022, the authors show that fewer than half (48.5%) were taking any statin at presentation, that roughly 9 in 10 had an LDL-C ≥55 mg/dL and 4 in 5 ≥70 mg/dL, and that combination lipid-lowering therapy was prescribed to only 5% at discharge. None of these figures improved measurably across the 5-year study period.^1^ The findings are not novel in kind; real-world lipid control has disappointed for years. Yet their specificity, and their anchoring to the index AMI, make them an unusually clarifying snapshot of where CV prevention breaks down.

The first failure occurs upstream of the infarct. Two-thirds of the cohort had no prior atherosclerotic cardiovascular disease (ASCVD) diagnosis, and most were statin-naive despite already qualifying for treatment: 68.6% met 2018 guideline indications, and, in the paper’s most provocative observation, 89% would have met indications under the 2026 American College of Cardiology/American Heart Association guideline using the PREVENT equations.^1^^,^^2^ It is tempting to read this as misfortune: the asymptomatic patient who felt well until the day he did not. The larger literature forbids that comfort. In a binational analysis of more than 9 million person-events, Lee at al^3^ found that, before their first cardiovascular disease (CVD) event, more than 99% of individuals had at least 1 traditional risk factor above its optimal level, and 90% to 95% had at least 1 that was frankly elevated. The “risk-factor–less” AMI is largely an artifact of missed measurement. What Essa et al^1^ document, then, is not the intrinsic unpredictability of AMI but the systematic failure to identify and treat a risk that was there to be found.

The second failure occurs at the event itself, and here the picture is paradoxical. Discharge prescribing of high-intensity statins was excellent (82% overall), evidence that clinicians reliably execute the familiar, single-step decision. But combination therapy remained vanishingly rare (5%), and among patients with established ASCVD and an LDL-C ≥55 mg/dL already taking a statin, 61% left the hospital with no intensification of any kind.^1^ This is the more troubling gap, because the index hospitalization is among the highest-yield teachable moments in medicine, and because the evidence for acting on it is unambiguous. Randomized trials have shown that adding ezetimibe or a proprotein convertase subtilisin/kexin type 9 inhibitor after an acute coronary syndrome lowers LDL-C further, favorably remodels coronary plaque, and reduces recurrent CVD events.^4^^,^^5^ Doubling a statin dose yields only about 6% additional LDL-C lowering; adding a second agent yields substantially more. And although the cost and prior-authorization friction surrounding proprotein convertase subtilisin/kexin type 9 inhibitors offer a partial alibi, ezetimibe is generic, oral, inexpensive, and well tolerated, so its near-absence cannot be explained by economics alone.

Why, then, does a field that agrees on the science so consistently fail at the bedside? The reflexive explanations (insufficient education, clinician indifference, patient nonadherence) do not survive contact with these data. Statin prescribing at discharge was quite frequent; clinicians plainly know the guideline and act on it when the action is simple. The gap opens precisely where the decision becomes multivariable: whether, when, and how to add a second agent against a target that depends jointly on ASCVD status, baseline LDL-C, calculated risk, comorbidity, and tolerability. Excellent execution of the one-step decision alongside collapse at the multistep one is the signature not of ignorance but of cognitive load.

That observation deserves to be taken seriously as a mechanism rather than a metaphor. Human working memory and multivariable integration are bounded, and the number of variables a clinician can jointly integrate in a single decision is small. As Morris^6^ has argued, the information a modern clinical decision demands routinely exceeds that limit, so even expert clinicians apply best evidence inconsistently. Guidelines, meanwhile, have moved in the opposite direction, evolving from simple threshold rules into dense, conditionally branched decision networks. The very 2026 dyslipidemia guideline that Essa et al^1^ invoke to demonstrate expanded eligibility illustrates the point: it widens *who* should be treated while simultaneously raising the cognitive cost of *each* treatment decision, folding PREVENT-based risk, renal function, and multiple modifiers into a single recommendation that an unaided clinician must resolve within a 15-min encounter.^2^ Expanding the numerator of the eligible without lightening the denominator of the decision is a recipe for exactly the intensification gap the authors observe. Seen this way, the flat 5-year trend is less a failure of will than a predictable consequence of asking human cognition to do more than it was built to do, a diagnosis Kishore and Kocher^7^ reach independently for hypertension, where control has stalled near 50% despite equally simple and effective therapy, and which they attribute to “a failure not of science but of system design.”

That framing matters because the lipid gap is not an isolated lesion. Across cardiometabolic care, control has plateaued for a decade: among adults with hypertension, concurrent diabetes and hyperlipidemia now cluster in more than 1 in 5, with only about a quarter achieving control of all 3, a figure unchanged in more than 10 years even as hypertension-related mortality has risen.^8^ Essa et al^1^ have given us the lipid-specific instance of a general phenomenon. The uniformity of the failure across risk factors, guidelines, and years is itself the strongest evidence that the problem is structural.

If the deficit is one of delivery rather than discovery, the remedies are structural too, and 2 are already supported by evidence. The first is to stop asking clinicians to traverse high-dimensional guidelines from memory and instead embed that logic in the workflow, so that well-designed clinical decision support precomputes the recommendation (statin intensity, target, next agent) and presents it for acceptance or override, rather than adding another alert to an already saturated screen.^6^ The second is to relocate execution of chronic, guideline-intensive management onto multidisciplinary teams (clinical pharmacists, advanced-practice clinicians, and nurses) operating within algorithm-coordinated protocols. Where this has been tested it works: pharmacist-led, protocol-driven management has repeatedly achieved guideline-concordant control well beyond usual physician care.^9^^,^^10^ These are not aspirations but the same class of intervention that carried Kaiser Permanente Northern California to 90% hypertension control while national rates stagnated.^7^ What they share is a refusal to treat implementation as a problem of individual diligence.

The analysis by Essa et al^1^ carries the limitations they candidly name: a single academic center whose resourced environment likely understates the gaps elsewhere, prescription data that cannot confirm adherence, and no window onto postdischarge titration that might yet close part of the deficit. None of these soften the central signal. If anything, a tertiary center with an engaged cardiology service represents a best case, and the best case is sobering.

The enduring value of this paper is that it shows the same patient failed twice: once in the years before the infarct, when treatable risk went unidentified, and again at the infarct itself, when the chance to intensify was let pass. Neither failure reflects a gap in what medicine knows. Both reflect a gap in how reliably that knowledge is delivered through fallible, time-pressed, cognitively finite clinicians working in systems not yet designed to help them. Closing it will require building those systems: defaults, algorithms, and teams that make the guideline-concordant choice the path of least resistance. The science of lipid lowering is, for now, essentially finished. The engineering of its delivery has barely begun.

## Funding support and author disclosures

Dr Lavie has served as a speaker and consultant for Amgen on their PCSK9i and for DSM and GOED on omega-3 supplements. Dr Milani has reported that he has no relationships relevant to the contents of this paper to disclose.
